# Supplementary material for: A Missense Variant in PDK1 Associated with Severe Neurodevelopmental Delay and Epilepsy
Source: Biomedicines. 2022 Dec 7;10(12):3171. doi: 10.3390/biomedicines10123171 (PMC9775741; doi:10.3390/biomedicines10123171)
Supplement: Supplementary file 1 [file biomedicines-10-03171-s001.zip › biomedicines-1974234-supplementary.pdf]

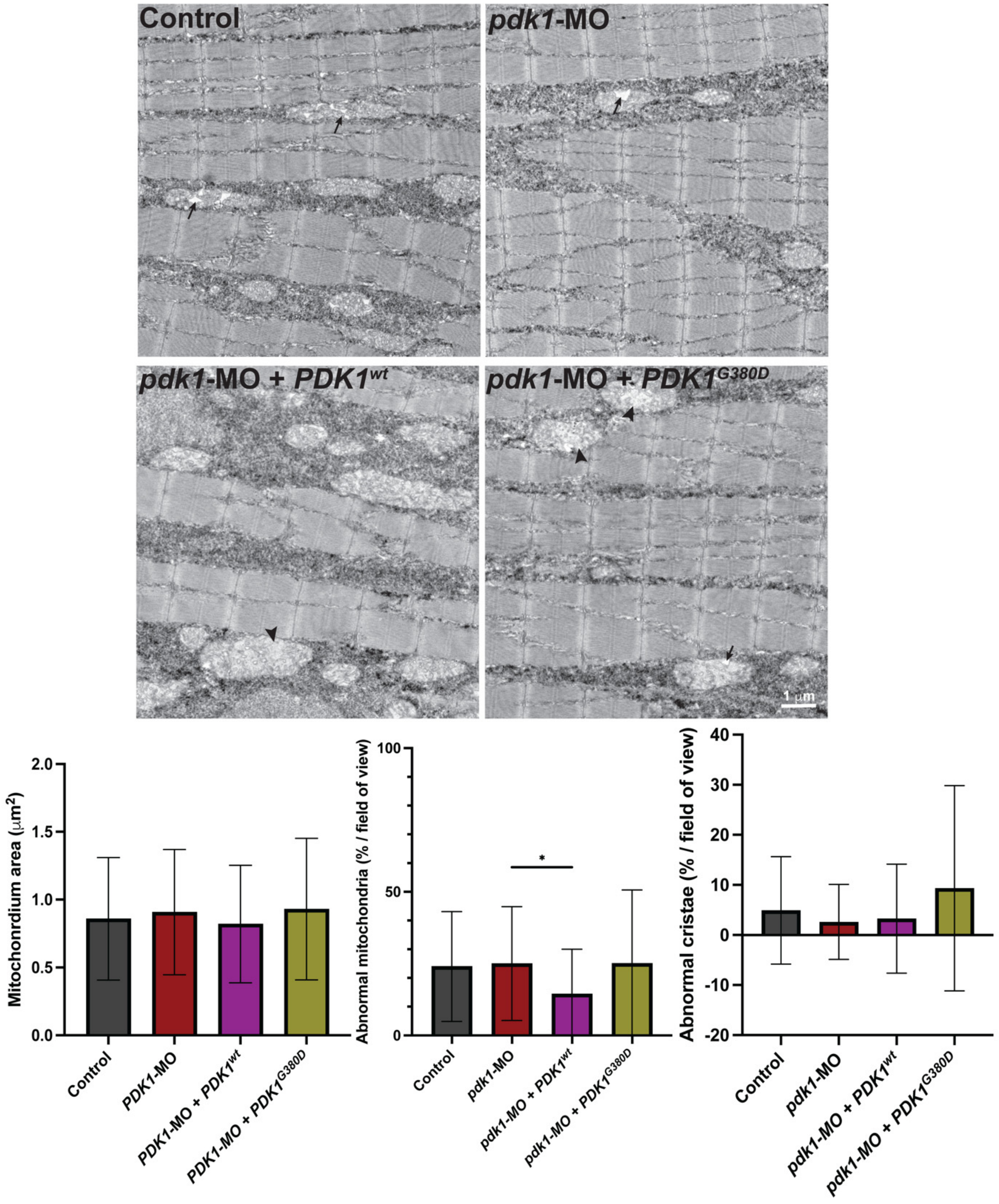

Figure S1. Mitochondria structure is not affected by mutant PDK1. Electron micrographs in the skeletal muscle show no significant differences in the mitochondria size nor structure between embryos from the different experimental groups. Some abnormalities in mitochondria were observed, such as vacuole-like structures (arrows) but were not consistently different between groups and we suspect they are a result of technical issues in the processing of the samples (*p = 0.0305). Biologically relevant phenotypes such as defects in cristae (arrowheads) were found but not significantly different between groups (n = 5 embryos per group and 10 electron micrographs per embryo).

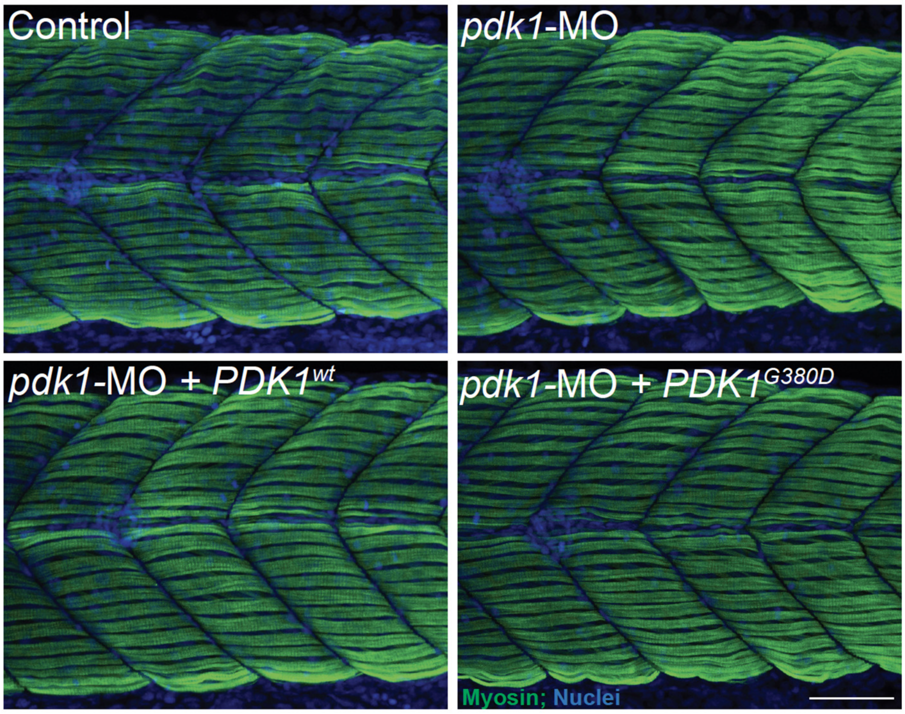

Figure S2. Mutant PDK1 does not affect muscle integrity. Labelling of zebrafish embryos for Myosin (in green) confirms the absence of any structural defect in the skeletal muscle in the embryos of any experimental group. Scale bar: 100 µm.
